# Oral polychemotherapy to induce remission in newly diagnosed type 2 diabetes: results from a randomized controlled trial

**DOI:** 10.1007/s40618-026-02930-3

**Published:** 2026-07-27

**Authors:** Alessandra Dei Cas, Raffaella Aldigeri, Giuseppe Maglietta, Emanuela Balestreri, Monia Garofolo, Giuseppe Daniele, Giuseppe Penno, Maddalena Trombetta, Alessandro Csermely, Monica Antonini, Angela Vazzana, Valentina Moretti, Sara Flamigni, Valentina Spigoni, Gloria Cinquegrani, Federica Fantuzzi, Marcello Monesi, Paolo Di Bartolo, Uberto Pagotto, Caterina Caminiti, Riccardo C. Bonadonna

**Affiliations:** 1 Department of Medicine and Surgery, University of Parma, Parma, Italy; 2 Endocrinology and Metabolic Diseases, Azienda Ospedaliero-Universitaria of Parma, Parma, Italy; 3 Clinical & Epidemiological Research Unit, University Hospital of Parma, Parma, Italy; 4 Department of Clinical and Experimental Medicine, University of Pisa, Pisa, Italy; 5 Department of Medicine, University of Verona, Verona, Italy; 6 Division of Endocrinology, Diabetes and Metabolic Diseases, Department of Medicine, Azienda Ospedaliera Universitaria Integrata di Verona, Verona, Italy; 7 Division of Endocrinology and Prevention and Care of Diabetes, IRCCS Azienda Ospedaliero-Universitaria di Bologna, Policlinico di Sant’Orsola, Bologna, Italy; 8 Primary Care Department, Unit of Diabetology, University Hospital of Ferrara, Ferrara, Italy; 9 Local Health Authority of Romagna, Ravenna, Italy; 10 Department of Medicine and Surgery, University of Parma, and Endocrinology and Metabolic Disease Azienda Ospedaliero-Universitaria of Parma, Via Gramsci 14, Parma, 43126 Italy

**Keywords:** Type 2 diabetes, Remission, Multidrug therapy, Randomized trial

## Abstract

**Purpose:**

To assess the efficacy of a four-drug regimen (POLYCHEM) vs. standard diabetes care (SDC) in inducing remission in patients with newly diagnosed type 2 diabetes.

**Methods:**

A multicenter, open-label, pragmatic, phase III, randomized clinical trial (NCT04271189) was conducted in 6 Italian Diabetes Outpatient Clinics. Patients were randomized (visit 1) to receive POLYCHEM or SDC for 16 weeks, after which, if there was regression of diabetic hyperglycemia (visit 2), drug therapy was suspended. The primary endpoint was remission (HbA1c < 6.5%; 48 mmol/mol) assessed at least 12 weeks (visit 3) after ceasing any glucose-lowering pharmacotherapy.

**Results:**

108 patients (80% males, age: 58 ± 9 yrs), were enrolled in the study, of whom 60 were randomized to POLYCHEM and 48 to SDC treatment. After 16 weeks, 54 (90.0%) and 37 (77.1%) patients achieved regression of diabetic hyperglycemia in the POLYCHEM and SDC arm, respectively (*p* = 0.002). At visit 3, 23 (38.3%) and 21 (43.8%) patients achieved diabetes remission in the POLYCHEM and SDC arm, respectively (*p* = 0.59). The difference between the upper 95% CI in POLYCHEM (50.9%) and the lower 95% CI in SDC (30.4%) of diabetes remission was 20.5%.

**Conclusion:**

A four-drug regimen with minimal to null weight-lowering effect is not superior to current SDC in achieving remission in patients with newly diagnosed type 2 diabetes. If a superiority of POLYCHEM over SDC exists, it is expected to be ≤ 20.5%.

## Introduction

Diabetes affects over 600 million adults worldwide, and projections indicate a further rise to 853 million (13%) by 2050 [1]. This epidemic, largely driven by westernized lifestyles and obesity, is associated with severe complications and reduced quality of life, and contributes to nearly one in ten deaths worldwide. Beyond clinical care diabetes already accounts for 12% of global health expenditure, placing a heavy burden on healthcare systems and societies. Within this scenario type 2 diabetes (T2D) constitutes over 90% of all cases, underscoring the urgency of developing innovative, scalable and sustainable strategies for prevention, management and - critically – remission [2]. Remission, as defined by the latest consensus criteria as HbA1c to < 6.5% over a period of at least 3 months in absence of glucose-lowering pharmacotherapy or at least 6 months after the lifestyle intervention [3, 4], has emerged as a clinically relevant and doable therapeutic goal. When achieved early, remission can delay or even prevent T2D-related complications, with benefits comparable to preventive strategies, while substantially reducing disease burden. To date, metabolic surgery [5, 6] and (very) low-calorie diets (VLCDs) [7–9] have consistently been shown to induce sustained T2D remission. These approaches have demonstrated that remission is achievable through substantial body weight reductions (> 15–20%) [10–12]. More recently, comparable weight loss obtained through treatment with Glucagon-like peptide 1 (GLP-1) receptor agonists [13], dual (GLP-1 receptor and Glucose-dependent Insulinotropic Polypeptide -GIP- receptor) [14, 15] and triple (GLP-1, GIP and glucagon receptors) [16] ligands, and combined GLP-1 receptor and amylin/calcitonin receptor [17] agonists has been associated to falls in HbA1c levels to ≤ 6.5% (48 mmol/mol) in high proportions of patients (50–80%). However, in people with obesity, upon treatment discontinuation, body weight rebounds and glycemic control deteriorates, suggesting that remission may be not permanent in the absence of incretin based therapy [18].

Other interventions aiming at diabetes remission have also been linked to significant weight loss. An intensive strategy combining lifestyle intervention, metformin and insulin glargine/lixisenatide achieved higher remission rates than standard care [19]. In contrast, a 16-wk intervention with insulin degludec/liraglutide failed to reach a statistically significant improvement in rates of diabetes remission vs. standard care, but this difference paralleled a minimal weight loss difference (< 2 kg) [20].

As to weight-loss independent interventions, in individuals with newly-diagnosed T2D, the initiation of insulin therapy reportedly induced remission, apparently by restoring beta-cell secretory function, presumably through glucose normalization and reduction in glucotoxicity [21]. Interventions with oral anti-diabetes drugs featuring glucose-lowering, but modest or no weight-lowering, action have provided mixed results [22, 23]. However, none of these approaches has tested the simultaneous use of four agents targeting distinct aspects of T2D pathophysiology at its early stage.

In this context, we conducted a proof-of-concept study to assess whether a four-drug regimen, engaging multiple glucose-lowering mechanisms, but with limited or no weight-lowering potential, could induce regression of diabetic hyperglycemia (HbA1c < 6.5%, 48 mmol/mol) and, after 3-month treatment suspension, achieve diabetes remission in a clinically meaningful higher proportion of patients compared with standard diabetes care (SDC) in people with newly diagnosed T2D.

## Methods

### Study design

This is a multicenter, open label, pragmatic, phase III, randomized 1:1 controlled clinical trial, designed to compare the efficacy of early initiation of a new therapeutic strategy of four drugs (POLYCHEM) vs. conventional care (SDC), in subjects with newly diagnosed T2D (NCT04271189). The study involved six Italian Diabetes Outpatient Clinics (Parma, Ferrara, Ravenna, Bologna, Verona and Pisa) and was conducted according to the guidelines of the Declaration of Helsinki and approved by the local Ethics Committee “Comitato Etico dell’Area Vasta Emilia Nord” with EUDRACT registration no. 2018-000833-12. Written informed consent was obtained from all subjects involved in the study. The planned duration of the study was two years, but an extension was granted due to the COVID-19 pandemic. Therefore, the enrolment period spanned from October 2020 to July 2023. The protocol was amended in January 2022, after the publication of the new consensus report which revised remission criteria [4]. These new criteria significantly differed from the previous ones and included the achievement of HbA1c < 6.5% (< 48 mmol/mol) for 12 weeks, in the absence of any glucose lowering pharmacologic treatment [4]. In response to the updated definition and to operational requirements, the study design was modified from the initially planned two-phase adaptive approach (phase IIb-phase III) with 2:1 ratio (POLYCHEM: SDC) to a phase III design with 1:1 allocation ratio (amendment approved on 7 April 2022).

### Study population

Inclusion criteria included age ≥ 35 and ≤ 75 years, newly diagnosed (within 6 months) T2D according to American Diabetes Association (ADA) criteria [24], negative anti-GAD antibodies, C-peptide ≥ 0.3 nmol/l, body mass index (BMI) ≥ 23 and ≤ 40 kg/m^2^, HbA1c ≤ 10% (86 mmol/mol), absence of diabetic retinopathy. Main exclusion criteria were: current or history of cancer within the last 5 years, acute cardiovascular event in the previous six months, Class III-IV heart failure according to the New York Heart Association (NYHA) classification, corticosteroid-based drugs or immunosuppressive medications, estimated creatinine-clearance (eGFR) < 45 mL/min/1.73 m^2^ assessed by with CKD-EPI formula, severe organ failure, multi-injection insulin therapy, contraindications to any of the study drug, as well as pregnancy or lactation. Women of childbearing age were eligible only if on birth control.

### Study intervention

Patients were randomized to either SDC or “POLYCHEM” (intervention arm). The latter consisted in the combination of four drugs: metformin, extended-release formulation, titrated to 2000 mg/day, pioglitazone 30 mg/day, sitagliptin 100 mg/day, and empagliflozin 10 mg/day. SDC consisted in the conventional treatments provided at each center.

Patients with newly diagnosed T2D were randomized at baseline (V1) to receive POLYCHEM or SDC for 16 weeks. At the end of this period (V2), based on the original protocol version (simultaneous nondiabetic values of fasting glucose < 126 mg/ml, 2-hour glucose after OGTT < 200 mg/ml and HbA1c < 6.5%) or according to the amended protocol [HbA1c < 6.5% (< 48 mmol/mol)]) diabetic hyperglycemia was assessed. If regression of diabetic hyperglycemia [HbA1c < 6.5% (< 48 mmol/mol)] was achieved, anti-diabetes therapy was discontinued irrespective of the randomization arm and patients were monitored for an additional 52 weeks (in the first protocol version) or 12 weeks (amended protocol) on no pharmacological therapy (V3). Conversely, if diabetic hyperglycemia regression was not achieved at V2, anti-diabetes therapy was modified irrespective of the randomization arm, according to clinical guidelines at the discretion of the treating physician. Furthermore, at V2, investigators could decide to stop the POLYCHEM treatment at any time at their discretion, for safety or intolerability reasons, based on laboratory parameters or clinical events. At 28 weeks (V3) HbA1c was re-assessed, to test diabetes remission [< 6.5% (48 mmol/mol)] after ceasing any glucose-lowering pharmacotherapy for 12 weeks.

### Objectives and endpoints

The primary objective was to determine whether POLYCHEM was superior to conventional care in inducing diabetes remission defined as HbA1c < 6.5% (48 mmol/mol) for at least 12 weeks after cessation of any glucose-lowering intervention (2021 Consensus Report) [3] which substantially revised the 2009 criteria [4]. The primary endpoint is the difference in diabetes remission rates [HbA1c < 6.5% (48 mmol/mol), assessed at least 12 weeks (V3) after ceasing any glucose-lowering pharmacotherapy, between study groups. Secondary endpoints include hyperglycemic regression (at 16 weeks) and changes in HbA1c and body weight throughout the study.

Planned secondary objectives, including quality of life and direct costs (see clinicaltrials.gov registration NCT04271189), are not addressed in this paper and will be reported in further publication. The primary end-point was evaluated at least 12 weeks after drug discontinuation for all enrolled patients.

### Sample size

To evaluate the efficacy of POLYCHEM in terms of the percentage of patients achieving remission at 28 weeks, sample size was calculated by using a one-sided 5% lower limit confidence intervals for the difference between two proportion test.

A between-group difference of 15% was assumed, with the requirement that the lower bound of the confidence interval should not be less than 5%. Accordingly, a total of 92 patients per group (*n* = 184) was required.

The primary efficacy analysis was conducted according to the Intention-To-Treat (ITT) principle. Univariable and multivariable logistic regression models were implemented to describe the association between treatment and the primary endpoint.

### Randomization and Blinding

Random allocation sequences were generated stratifying per center and using blocks of 2 or 3. Randomization lists were generated by the trial statistician by specifying a unique seed for each list and using the blockrand R package. Allocation concealment was ensured by storing lists in the centralized electronic Case Report Form (eCRF) developed by an external service using the OpenClinica platform. Blinding was not possible due to the process of treatment administration and clinical outcome assessment. In order to minimize bias the primary outcome represents an objective and reliable outcome (HbA1c).

### Statistical analysis

The analyses were conducted according to ITT, including all randomized participants in their originally assigned groups. The primary efficacy endpoint was analyzed using a two-proportion chi-square test to compare remission rates between the two arms. No missing data were recorded for primary or secondary outcomes. Baseline characteristics are presented as mean (Standard Deviation, SD) or median (Interquartile Range, IQR) for quantitative variables and absolute and relative frequencies for qualitative variables. Changes over time between treatment arms were assessed using mixed-effects models for repeated measures.

Univariate and multivariate logistic regression analyses were performed to identify variables associated with diabetes remission, including age, sex, baseline HbA1c, weight loss at 16 weeks, and statin therapy as a covariate. No data imputation was performed.

All statistical analyses were performed with R Statistical Software, V 4.3.0 and SPSS (IBM Statistics v.29).

## Results

### Study population

**Fig. 1:**
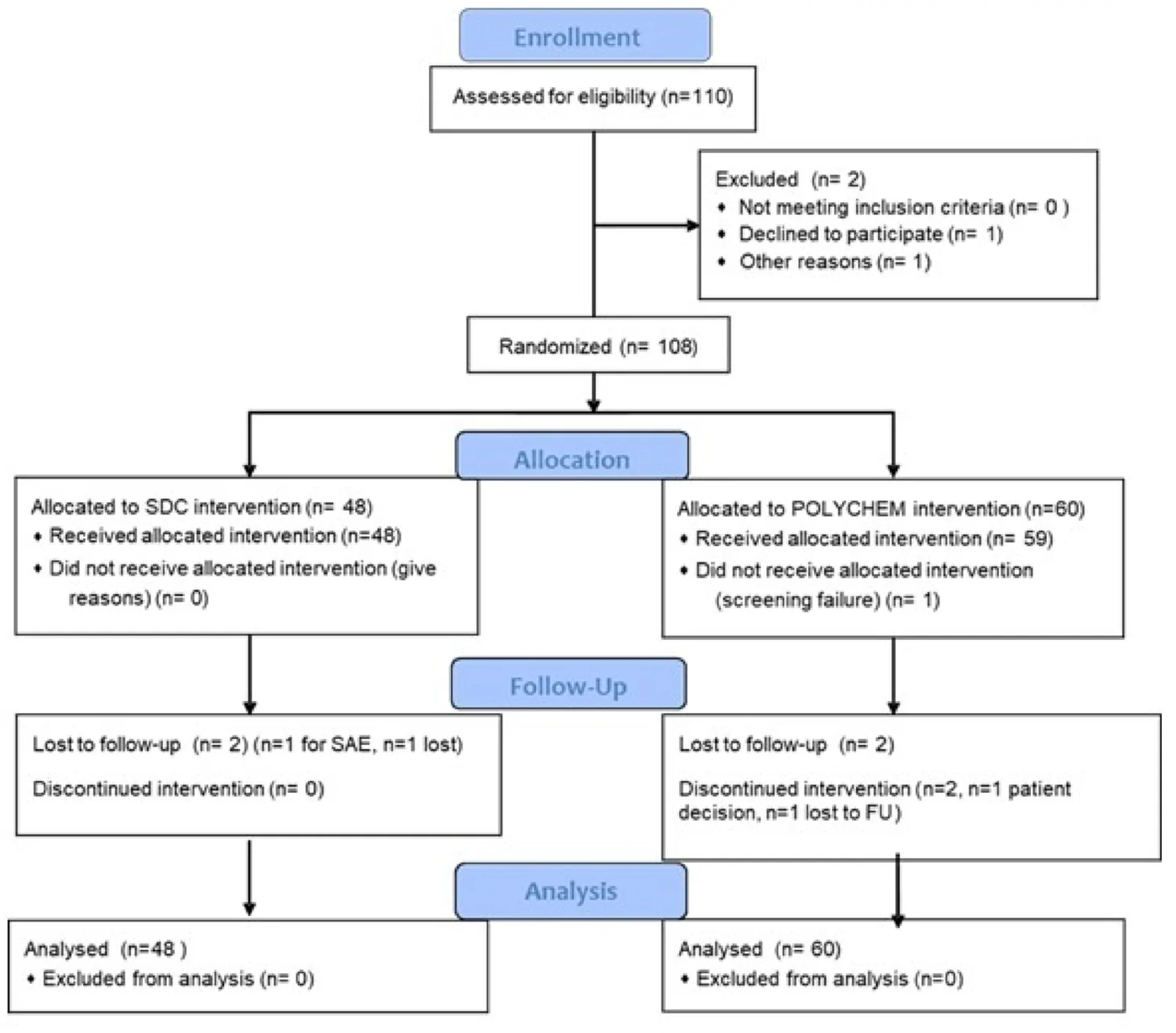
**Study flow diagram according to ITT analysis**

Overall, between October 2020 and July 2023, 110 patients with newly-diagnosed T2D were screened, of whom 108 (98.2%) were randomized, 60 (55.5%) to the POLYCHEM intervention and 48 (44.5%) to the SDC control arm (Fig. 1). Initially, the study followed a two-phase adaptive design (phase IIb-phase III) with 2:1 ratio (20 subjects have been randomized to POLYCHEM vs. 12 to SDC) and was subsequently amended to a phase III design with 1:1 allocation ratio (see Study Design).

During follow-up, two patients in the SDC arm (4.2%), and 2 (3.3%) in the POLYCHEM arm were lost at follow-up. One subject in the POLYCHEM arm was identified as screening-failure.

Schedule of enrolment, interventions, and assessments with number of subjects at different time points is presented in Table 1.

**Table 1:**
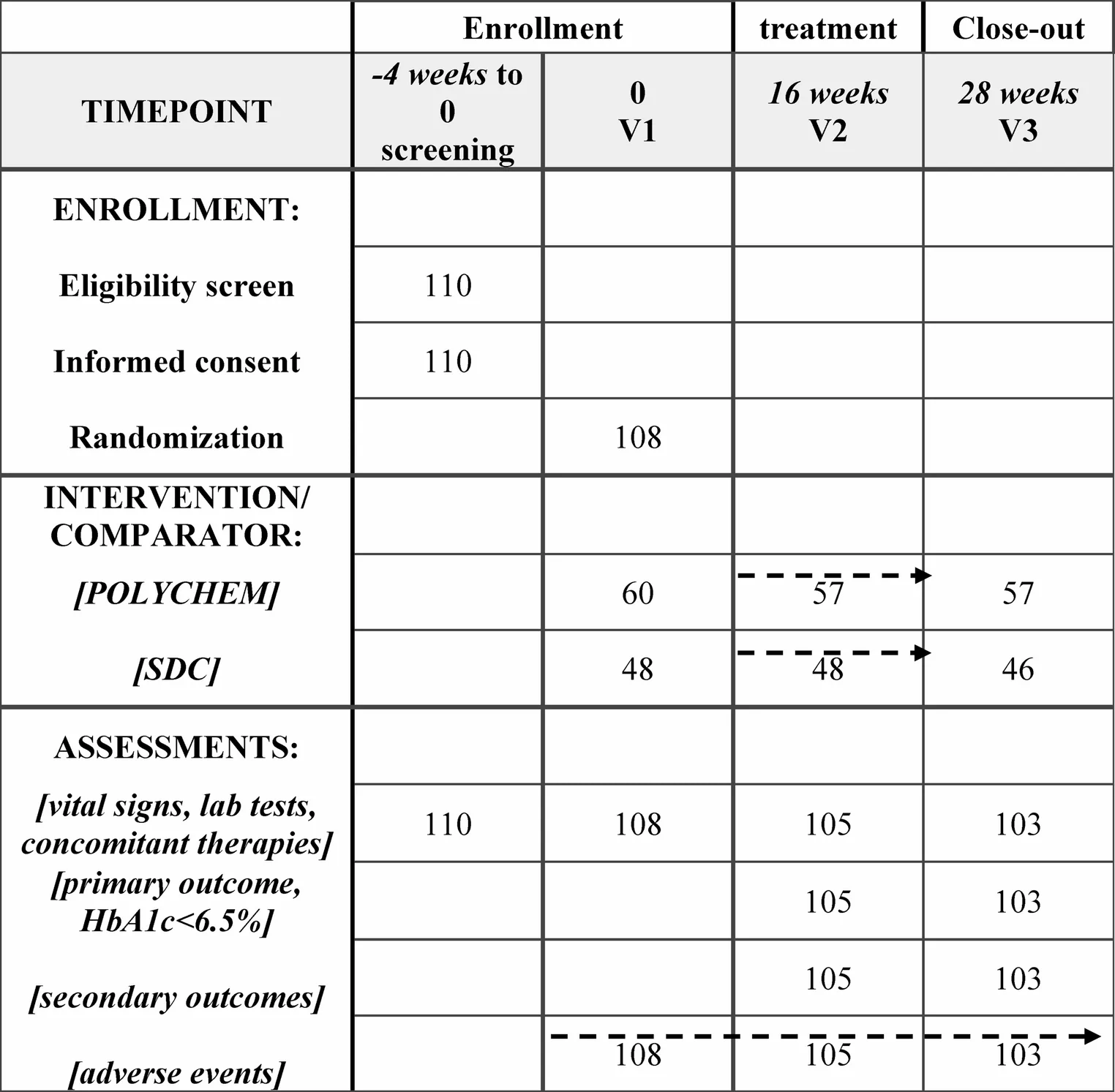
**Schedule of enrollment, interventions, and assessments with number of subjects at different time points**

Table 2 shows baseline demographic and clinical characteristics of participants which were similar between the two groups, as expected by the randomization procedure. Overall, patients were prevalently male (80%), with median age 58 years. Mean BMI was 29.9 kg/m^2^ and baseline HbA1c was 6.6% (49mmol/mol); almost 18% were active smokers. The prevalence of comorbidities, including hypertension, dyslipidemia, and previous cardiovascular disease (CVD) was statistically comparable between groups. No significant differences between arms were recorded among concomitant therapies and CVD history (Table 2).

**Table 2:** **Baseline Characteristics of the study population**

	**Overall, N = 108**	POLYCHEM, *N* = 60	SDC, *N* = 48
**Age (years)**	58 ± 9	60 ± 8	56 ± 9
**Female sex**	22 (20%)	13 (22%)	9 (19%)
**Ethnicity**,** caucasian**	108(100%)	60(100%)	48(100%)
**BMI (kg/m** ^**2**^ **)**	29.9 ± 4.8	29.6 ± 5.3	30.3 ± 4.1
**SBP (mmHg)**	134 ± 14	135 ± 14	133 ± 15
**DBP (mmHg)**	82 ± 11	82 ± 11	83 ± 11
**Waist circumference(cm)**	105.0 ± 11.5	104.9 ± 11.0	104.9 ± 12.2
**HbA1c (mmol/mol)**	49(44–55)	48(44–56)	49(43–55)
**HbA1c (%)**	6.6(6.2–7.2)	6.5(6.2–7.3)	6.6(6.1–7.2)
**Fasting plasma glucose (mg/dl)**	125(114–138)	124(110–140)	126(116–137)
**Fasting plasma glucose (mmol/l)**	6.94(6.33–7.67)	6.89(6.11–7.78)	7.00(6.44–7.61)
**Total cholesterol (mg/ml)**	175.6 ± 38.7	179.4 ± 36.2	170.8 ± 41.6
**HDL-C (mg/dl)**	47.6 ± 10.9	47.8 ± 11.2	47.5 ± 10.6
**LDL-C (mg/dl)**	101.7 ± 34.1	103.7 ± 31.8	99.1 ± 37
**TG (mg/dl)**	119.5(85.5-155.5)	131.5(89–157)	110(79.7-147.5)
**AST (U/L)**	23(20-28.5)	23(19–28)	24(20–32)
**ALT (U/L)**	29(21–40)	29(22.2–38)	26(20–41)
**Lipase (U/L)**	34(22–45)	36(26-48.5)	31(20–44)
**eGFR (ml/min)**	92.9 ± 15.3	92.1 ± 16.9	93.9 ± 13.1
**ACR (mg/g)**	9.1(4.2–20)	10(4.4–38.9)	7.3(4.1–13.3)
**Current Smoker**	19 (17.6%)	8 (13.3%)	11 (22.9%)
**Dyslipidemia**	65(60.2%)	38(63.3%)	27(56.3%)
**Hypertension**	55(50.9%)	30(50%)	25(52.1%)
**Previous cardiovascular event**	5(4.6%)	2(3.3%)	3(6.3%)

Data are given as n (%) for categorical variables and as mean ± SD or median (interquartile range) for continuous variables. Abbreviations HbA1c= glycated hemoglobin A1c, BMI= Body-Mass Index, SBP= Systolic blood pressure, DBP= Diastolic blood pressure, HDL-C= high-density lipoprotein-cholesterol, LDL-C = low-density lipoprotein-cholesterol, TG= triglycerides, AST= Aspartate Aminotransferase, ALT= Alanine Aminotransferase, eGFR= eGFR, estimated glomerular filtration rate, ACR= albumin to creatinine ratio

### Anti-diabetes therapy in the SDC arm

Glucose lowering drugs in the SDC arm at baseline (V1) and during follow-up (V2-V3) are reported in Table 3: among therapies at baseline 43 patients (89.6%) were on metformin, 18 (37.5%) on SGLT-2 inhibitors, 4 (8.3%) on DPP4 inhibitors, 5 (10.4%) were treated with basal insulin therapy and 4 (8.3%) with GLP-1 receptor agonist.

**Table 3:** **Glucose lowering drug in the SDC arm at baseline and during follow up**

Glucose lowering drugs in SDC arm	Baseline (V1)	16 weeks (V2)	28 weeks (V3)
**None**	2 (4.2%)	26 (54.2%)	26 (43.8%)
**1 oral** -metformin -SGLT2i	22 (45.8%) 21(43.7%) 1(2.1%)	8 (16.7%) 7 (14.6%) 1 (2.1%)	7 (14.6%) 6 (12.5%) 1 (2.1%)
**2 orals** -metformin+SGLT2i -DPP-4i+SGLT2i -metformin + DPP-4i	14 (29.2%) 13 (27.1%) 1 (2.1%) 0	8 (16.7%) 7 (14.6%) 0 1 (2.1%)	8 (16.7%) 7 (14.6%) 0 1 (2.1%)
**3 orals** -metformin + DPP-4i +SGLT2i	1 (2.1%) 1 (2.1%)	2 (4.2%) 2 (4.2%)	2 (4.2%) 2 (4.2%)
**1 oral + 1 subcutaneous** -metformin + GLP-1 RA -metformin+insulin -SGLT2i+insulin	6 (12.5%) 4 (8.4%) 1 (2.1%) 1 (2.1%)	3 (6.2%) 1 (2.1%) 1 (2.1%) 1 (2.1%)	3 (6.2%) 1 (2.1%) 1 (2.1%) 1 (2.1%)
**2 orals + 1 subcutaneous** -metformin+SGLT2i+insulin -metformin + DPP-4i +insulin -metformin+SGLT2i + GLP-1 RA	3 (6.2%) 2 (4.2%) 1 (2.1%) 0	1 (2.1%) 0 0 1 (2.1%)	1 (2.1%) 0 0 1 (2.1%)
**1 subcutaneous** -GLP-1 RA	0 0	0 0	1(2.1%) 1(2.1%)

Data are expressed as n (%). SGLT2i: sodium-glucose transport protein 2 inhibitors, GLP-1 RA: Glucagon-like peptide-1 receptor agonists, DPP-4i: Dipeptidyl peptidase-4 inhibitor

### Effect of the POLYCHEM on diabetes remission (primary endpoint)

**Fig. 2:**
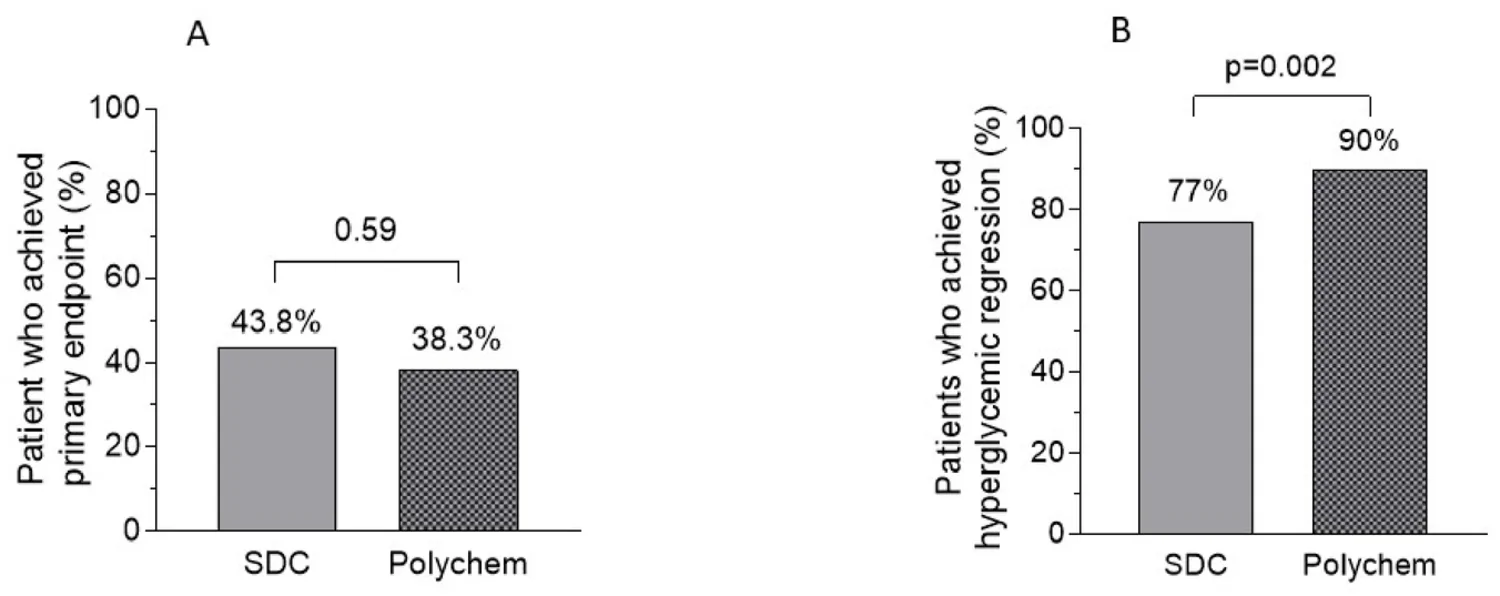
**Diabetes and hyperglycaemic remission rates**.** A**. Patients(%) who achieved diabetes remission at 28 weeks in the two study arms; **B**. Patients(%) who achieved hyperglycemia regression at 16 weeks in the two study arms

The POLYCHEM intervention exerted no significant effects on the rate of diabetes remission in newly diagnosed patients at weeks 28 as shown in Fig. 2A. At weeks 28 (V3) 38.3% (95% C.I.: 26.8–50.9) of the individuals in the POLYCHEM arm compared to 43.8% (95% C.I.: 30.4–57.8) in the SDC arm achieved the primary endpoint (*p* = 0.59). The difference between the upper 95% C.I. of POLYCHEM (50.9%) vs. the lower 95% C.I. of SDC (30.4%) was 20.5%.

### Effect of the POLYCHEM in the diabetic hyperglycemia regression

More patients achieved diabetic hyperglycemia regression (HbA1c < 48 mmol/mol at 16 weeks) in the POLYCHEM arm (90%) than in SDC (77%) arm (*p* = 0.002), as shown in Fig. 2B.

Table 4 shows the percentages of patients who achieved diabetic hyperglycemia regression at 16 weeks and diabetes remission at 28 weeks (HbA1c < 48 mmol/mol after treatment discontinuation) (4). Of note, 19 (31.6%) individuals in the POLYCHEM and 11 (22.9%) in the SDC arm achieving hyperglycemia regression continued anti-diabetes therapy due to the remission criteria used before protocol amendment which required the fulfilment of not only HbA1c < 6.5% (48 mmol/mol) levels but also the simultaneous normalization of fasting plasma glucose (FPG)< 126 mg/dl (7 mmol/l) and 2-hour glucose levels < 200 mg/dl (11.1 mmol/l) following a glucose tolerance test 75 g.

**Table 4:** **Percentages of patients who achieved the primary endpoint and the three relative necessary conditions**

Arm	Diabetic Hyperlycaemia regression: patients with HbA1c < 48 mmol/mol at 16 weeks	Patients who discontinued antidiabetic treatments during week 16–28	Patients who maintained HbA1c < 48 mmol/mol independently of anti-diabetes therapy cessation	DIABETES REMISSION at 28 weeks
**POLYCHEM**	54/60	35/60	35/60	23/60
	90.00%	58.37%	58.33%	38.33%
**SDC**	37/48	26/48	33/48	21/48
	77.08%	54.2%	68.75%	43.75%

### Effect of the POLYCHEM on HbA1C and body weight

**Fig. 3 HbA1c and body weight during the study period.**
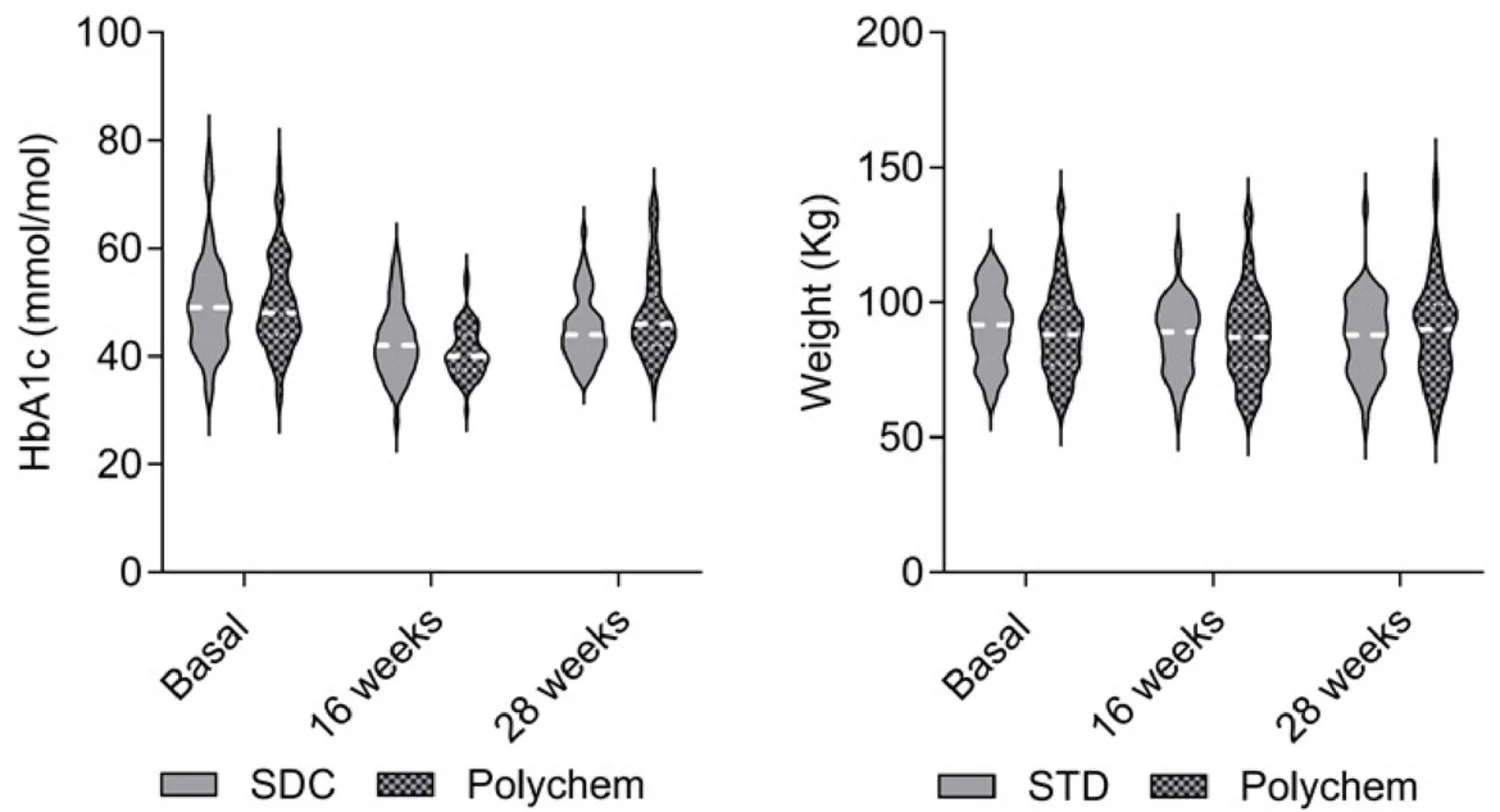
**A**. HbA1c (mmol/mol) and **B**. Body weight (kg) values in the two study arms at baseline and during follow-up

Figure 3 depicts changes in HbA1c (A) and body weight (B) during the study period in the two arms. At randomization, mean HbA1c values were identical in the two groups (6.6%; 49.7 mmol/mol) and decreased over the first 16 weeks (V2) in both arms, with a non-significant difference in favor of the POLYCHEM arm (∆-1.8, *p* = 0.24). At the second follow-up visit (28 weeks) (V3), HbA1c gradually increased in both arms, significantly more in the POLYCHEM arm (∆+4.8 *p* < 0.001). In the generalized linear model (GLM) model a significant time decrease in HbA1c was present (*p* < 0.001) without treatment effect (*p* = 0.93).

At baseline (V1), body weight was slightly, but not significantly higher in the SDC arm (+ 1.9 kg; *p* = 0.53), at 16 weeks body weight decreased in both arms without significant differences (*p* = 0.21), at 28 weeks (V3) a significant increase in body weight was observed in the POLYCHEM arm vs. SDC (+ 1.28 kg; *p* = 0.02). Similarly, in the GLM model a significant time*treatment effect was observed (*p* < 0.02) with a significantly greater reduction of weight in subjects randomized to SDC as compared to POLYCHEM arm.

Changes in glucose and other clinical parameters at different time points during the study are shown in Table 5. No significant differences were seen between treatment arms.

**Table 5:** **Glucose and clinical parameters at different time visits**

	POLYCHEM			SDC			*p*-val
	Baseline (V1)	16 weeks (V2)	28 weeks (V3)	Baseline (V1)	16 weeks (V2)	28 weeks (V3)	
	*n* = 60	*n* = 57	*n* = 57	*n* = 48	*n* = 48	*n* = 46	
HbA1c (mmol/mol)	48(44–56)	40(38–44)	46(42–52)	49.5(42.7–55.0)	42(38-47.2)	44(41-48.2)	0.94
FPG (mg/dl)	124(110–144)	113(104.4–129)	127(108–140)	126(116-137.2)	118(100.7-130.2)	122(108.7–1327)	0.64
Weight (kg)	89.1 ± 16.6	87.7 ± 16.7	89.6 ± 18.1	91.0 ± 13.9	87.6 ± 13.6	87.6 ± 14.9	0.81
BMI (kg/m^2^)	29.6 ± 5.23	29.5 ± 3.75	30.0 ± 4.28	30.3 ± 4.11	29.2 ± 3.93	29.2 ± 4.36	0.77
Waist circumference (cm)	105 ± 11.0	103 ± 10.8	104 ± 11.7	105 ± 12.2	101 ± 17.9	102 ± 12.3	0.46
SBP (mmHg)	135 ± 14.1	131 ± 15.9	135 ± 15.7	133 ± 14.8	132 ± 13.4	131 ± 14.0	0.43
DBP (mmHg)	82.2 ± 10.7	82.0 ± 12.1	82.8 ± 10.3	82.8 ± 10.6	83.5 ± 8.9	83.6 ± 8.7	0.65

Data are expressed as mean ± SD if normally distributed, or median (IQR) if skewed. P-value from generalized linear model for repeated measure. FPG, fasting plasma glucose; BMI, body mass index; SBP, systolic blood pressure; DBP, diastolic blood pressure

### Variables associated to diabetes remission

In the univariable logistic regression analysis, in the whole population, remission rates were negatively associated with higher baseline HbA1c [OR = 0.92 (95%CI: 0.87–0.97); *p* = 0.002] and FPG levels [OR = 0.94 (95%CI: 0.91–0.97); *p* < 0.001] and positively associated with a higher decrease in body weight at 16 weeks [OR = 1.14 (95%CI: 1.02–1.28); *p* = 0.02]. In a multivariate analysis, after adjusting for age, gender, baseline HbA1c values, weight reduction after 16 weeks, and statin therapy, only lower HbA1c values [OR = 0.91 (95%CI 0.86–0.97); *p* = 0.002] and weight loss [OR 1.16 (95%CI 1.04–1.30); *p* = 0.009] remained independent predictors of diabetes remission at 28 weeks in the whole population. When the analysis was conducted separately per randomization arm, in the POLYCHEM arm, baseline HbA1c values [OR = 0.83 (95%CI 0.73–0.94) *p* = 0.003] and weight reduction after 16 weeks [OR = 1.29 (95%CI 1.04–1.60) *p* = 0.02] were confirmed independent predictors of diabetes remission, whereas in the SDC arm no significant predictors were associated with the primary end-point (data not shown).

### Adherence to therapy

The mean percentage of compliance to the POLYCHEM therapy was 96.1% (SD = 7.9%). Two subjects (3.3%) randomized to POLYCHEM did not take therapy because of intolerance (non-specific symptoms such as such as fatigue and malaise). In the SDC arm (Table 6) three subjects (6.3%) decreased the dosage of metformin due to gastrointestinal side effects.

**Table 6:** **Distribution of adverse events (AE) in study participants**

Overall (*n*, %)	POLYCHEM (*n*, %)	SDC (*n*, %)	*p*-value
**40**,** 37%**	25, 41.7%	15, 31.3%	0.26
**Severity**			
**-mild** **-moderate** **-severe**	21, 84% 4, 16% 0	11, 73% 1, 7% 3, 20%	0.24
**Causality** **-not correlated** **-possible/probable**	16, 64% 9, 36%	12, 80% 3, 20%	0.33
**Type of AE**: **-Gastrointestinal**	12, 20%	5, 10%	0.36
**-Urogenital**	7, 12%	4, 8%	0.93
**-SARS-COV2 infection**	3, 5%	3, 6%	0.49
**-Respiratory tract infection** **-Dizziness** **-Low back pain** **-Paresthesia** **-Hypertension**	6, 10% 1, 2% 2, 3% 1, 2% 1, 2%	0 3, 6% 2, 4% 1, 2% 1, 2%	0.04 0.10 0.60 0.70 0.70

Data are given as n (%)

### Adverse events

Over the 28-weeks’ study period, 40 patients, 25 in POLYCHEM (41.7%) and 15 in SDC (31.3%) experienced adverse events, as summarised in Table 6, without significant differences. Three serious adverse events (SAEs) occurred in the SDC arm and included prostate cancer, acute cystopyelitis and hospitalization for road accident. The most common reported events were gastrointestinal (*n* = 17), urogenital (*n* = 11), SARS-COV2 infections (*n* = 6), and respiratory infection with or without fever (*n* = 6). Most of them were classified as mild adeverse events (*n* = 32/40, 80%) and not related to experimental drugs (*n* = 28/40, 70%). In the POLYCHEM arm 6 subjects (10%) reduced the dosage of metformin due to gastrointestinal side effects, 3 (5%) discontinued empagliflozin because of urogenital infections while 2 (3.3%) permanently discontinued all drugs.

## Discussion

Given the growing interest in diabetes remission as a therapeutic goal, this proof-of-concept trial was designed to test whether a fully oral polypharmacological regimen (sitagliptin + metformin + empagliflozin + pioglitazone) administered over 16 weeks was superior to SDC in achieving remission in newly diagnosed T2D. Remission rates were comparable between the two groups (38.3% in POLYCHEM vs. 43.8% in SDC), and no statistically significant differences in body weight were observed. These findings suggest that pharmacologically induced remission is feasible through weight-independent mechanisms, although the POLYCHEM strategy did not demonstrate superiority over SDC.

Dietary interventions [7–9] and bariatric surgery [25–28] have consistently proven effective in inducing durable diabetes remission, with weight loss serving as the primary mechanism underlying glucose normalisation. In bariatric surgery, additional biological factors -including changes in gut microbiota, bile acid metabolism, and the secretion of appetite-modulating peptide hormones- may further contribute to remission [29]. In contrast, remission in our study occurred in the absence of clinically meaningful (i.e. ≥ 5%) weight reduction, suggesting the existence of alternative mechanisms of metabolic recovery.

Beyond lifestyle and surgical approaches, early intensive pharmacological strategies at diagnosis have also been investigated. Early intensive insulin therapy in patients with recent-onset T2D improve β-cell function [30–32] and induce prolonged remission, likely through rapid correction of glucotoxicity, compared with treatment with oral anti-diabetes drugs. More recently, the combination of insulin pump therapy with Low-Calorie Diet (LCD) intervention was associated with improved remission rates in patients with newly diagnosed type 2 diabetes compared with standard care [33].

The Remission Evaluation of Metabolic Intervention in Type 2 Diabetes (REMIT) pilot trial showed that a polypharmacological approach over 16 weeks [metformin + acarbose + insulin glargine] was more effective in inducing normoglycemia and remission compared to standard care [41% vs. 14%] [22]. Similarly, the REMIT-dapa trial [34] and REMIT-sita trial [35] evaluated the efficacy of polypharmacological regimens with insulin glargine + metformin + dapagliflozin and insulin glargine + metformin/sitagliptin, respectively, vs. standard care. Both regimens, maintained for 12 weeks, and both demonstrated superior remission rates [HbA1c ≤ 6.5% (48 mmol/mol)] and reduced relapse compared to standard care.

To our knowledge, the present study is the first randomized trial testing a fully oral, multi-target regimen in newly diagnosed T2Ds using formal remission criteria, specifically addressing whether remission can be achieved without insulin-based strategies or clinically meaningful weight loss.

While remission rates in our study were similar between the intervention arm compared to the SDC arms (38.3% vs. 43.8%), the overall remission frequency in both arms (≈ 40%) was markedly higher than the spontaneous remission typically reported in newly diagnosed T2D (≈ 4–6%) [36], suggesting that early pharmacological intensification provided in outpatient specialist clinics may be a viable strategy to reverse hyperglycemia.

Importantly, the SDC arm in this study was not protocolized and reflected physician-driven therapeutic decisions across centers. While this pragmatic design enhances external validity, it may have introduced heterogeneity in treatment exposure. Notably, the recruitment period overlapped with evolving treatment recommendations and increasing adoption of early combination therapies in clinical practice. As a result, a substantial proportion of patients in the SDC arm received multidrug regimens early in the disease course. Consequently, the SDC arm likely functioned as an active comparator rather than a traditional stepwise-care control group. In addition, a potential trial effect related to closer follow-up cannot be excluded and may have contributed to treatment intensification, thereby reducing the contrast between groups.

The potential mechanisms underlying remission in our study deserve specific consideration. The POLYCHEM regimen simultaneously targeted key pathophysiological mechanisms driving the onset and progression of T2D, including insulin resistance, β-cell dysfunction, and glucotoxicity. Such a multimodal approach may enhance β-cell preservation and improve insulin sensitivity independently of body weight.

However, in contrast to weight-loss based interventions, which remain the best performers of remission, as demonstrated by interventions such as (very) low-calorie diets [37] and bariatric surgery [38], this strategy was not associated with clinically meaningful weight reduction.

Consistently, in our study, no statistically significant differences in body weight were observed between the two treatment groups, although a modest increase (+ 1.2 kg) was noted in the POLYCHEM arm. This may have attenuated its efficacy as weight loss emerged as an independent predictor of remission in the multivariable analysis.

The clinical applicability of the POLYCHEM strategy should be interpreted within the current therapeutic landscape. Given its minimal effect on body weight, its role may be limited in patients in whom weight reduction is a primary goal, where incretin-based therapies are increasingly preferred. However, a fully oral, multi-target approach may be relevant in selected patients, such as those not overweight or with contraindications to incretin-based therapies, by targeting key pathophysiological mechanisms independently of weight loss. Nevertheless, in the absence of demonstrated superiority over standard care, our results should be considered as supportive of the feasibility of a proof-of-concept approach requiring further validation.

An additional aspect to consider is the relatively low baseline HbA1c in our cohort, close to the remission threshold, which may have contributed to the high remission rates observed in both arms and limits generalizability to patients with more severe disease. In the POLYCHEM arm, lower baseline HbA1c was associated with a higher likelihood of remission, suggesting greater effectiveness in individuals with milder diabetic hyperglycemia. These findings do not make one envisage a greater treatment effect at higher HbA1c levels, although this warrants further investigation.

The POLYCHEM strategy was well tolerated, supporting its potential as a viable therapeutic approach to be further evaluated and implemented in larger population-based studies. Nevertheless, the study did not demonstrate superiority of a 16-week, four-drug oral regimen over SDC in inducing remission, and the findings should be interpreted as exploratory.

We had designed the study with the goal of being able to capture an absolute difference in the proportion of diabetic remission of 15%. Owing to end of funding, the actual power of this study was an 80% chance of detecting an absolute difference of 25% in the proportion of diabetes remission at *p* < 0.05. Since the proportion of diabetes remission in the SDC arm was 43.8%, POLYCHEM should have induced almost twice more (68.8%) of diabetes remissions than it actually did (38.3%) for reaching statistical significance. Furthermore, the upper 95% confidence interval of the proportion of diabetes remission in the POLYCHEM arm was 50.9%, much lower than the value (68.8%) which would be expected to be statistically significant. Finally, and most importantly, the difference between the upper 95% C.I. of POLYCHEM (i.e. a data-based estimate of the highest possible effect of POLYCHEM) and the lower 95% C.I. of SDC (i.e. a data-based estimate of the lowest possible effect of SDC) in diabetes remission was 20.5%. Thus, we think that this study provides evidence that the superiority, if any, of POLYCHEM over SDC in inducing diabetes remission is ≤ 20.5%.

Our study presents some strengths namely the randomized design and the rigorous assessment of diabetes remission based on the standardized criteria established by international consensus statements and clinical guidelines. This contrasts with recent incretin-based trials [13, 14, 16], where HbA1c ≤ 6.5% was reported under ongoing pharmacological treatment, thereby not fulfilling the formal remission definitions [4].

Limitations include the relatively small sample size and the and the non-protocolized SDC arm, which may have introduced heterogeneity and influenced outcome comparisons. In addition, the lack of longer-term follow-up limits conclusions regarding the durability of remission.

## Conclusions

In conclusion, this randomized trial in outpatients diabetes clinics shows that a fully oral, insulin-sparing polypharmacological regimen with no weight-lowering effect can induce remission in a substantial proportion of patients with newly diagnosed T2D. However, it did not prove superior to standard care. These findings support early pharmacological intensification as a potential avenue for remission, but larger and longer-term trials are needed to define the role of oral polypharmacological remission-aimed regimens in the evolving therapeutic landscape of T2D.

## Data Availability

Some or all datasets generated during and/or analyzed during the current study are not publicly available but are available from the corresponding author on reasonable request.

## Declarations

### Clinical trial information

ClinicalTrials.gov number: NCT04271189.
